# Disseminated perforating necrobiosis lipoidica: A case report and literature review

**DOI:** 10.1002/ccr3.7991

**Published:** 2023-10-03

**Authors:** Mina Saber, Fatemeh Mohaghegh, Parisa Mohammadian

**Affiliations:** ^1^ Department of Dermatology Skin Diseases and Leishmaniasis Research Center, Isfahan University of Medical Sciences Isfahan Iran

**Keywords:** dermoscopy, diabetes mellitus, granulomatous disease, necrobiosis lipoidica, perforating dermatosis

## Abstract

**Key Clinical Message:**

Necrobiosis lipoidica is a rare cutaneous granulomatous disease that mainly affects diabetic patients. The perforating type of the disease is an uncommon variant that is resistant to therapy and can be easily identified using dermoscopy.

**Abstract:**

Perforating necrobiosis lipoidica (NL) is a rare NL variant that primarily affects patients with diabetes mellitus (DM). Dermoscopy helps to differentiate this type of disease. The disseminated form of perforating NL mainly occurs in the setting of DM. Here we present a case of disseminated perforating NL in a 24‐year‐old woman with type 1 DM.

## Graphical abstract

Disseminated perforating necrobiosis lipoidica occuring in the context of type 1 diabetes mellitus.

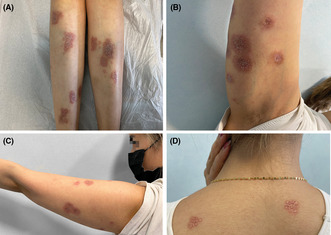

## 1 INTRODUCTION

Necrobiosis lipoidica (NL) is an uncommon chronic granulomatous disease that typically presents as ovoid plaques with a violaceous border and atrophic yellow center.^1^ It is typical for NL to occur in the pretibial region, but it can also occur in less typical and rare locations such as upper extremities, face, trunk, and scalp.^1^, ^2^ Controversy still exists concerning the pathogenesis of the disease; however, microangiopathy has been suggested as the primary cause of altered collagen seen in NL.^2^ A deposition of glycoprotein in the blood vessel walls of diabetic patients leads to microangiopathy and abnormal collagen fibrils, all considered end organ damage in diabetic patients.^3^

The incidence of NL in diabetic patients is 0.3%–1.2%. Perforating NL is an even rarer variant of the disease that was first described by Parra et al in 1977^4^ with less than 30 cases reported in the literature. Out of these cases, only five were disseminated, and all were found in patients with type 2 DM.^2^, ^4^, ^5^, ^6^, ^7^, ^8^ However, to the best of our knowledge, only one previous report has described the dermoscopic pattern of perforating NL.^8^ Herein, we present a case of disseminated perforating NL in a 25‐year‐old woman with type 1 diabetes mellitus along with its dermoscopic pattern. Then, we review the literature on disseminated forms of perforating NL.

## 2 CASE REPORT

**FIGURE 1:**
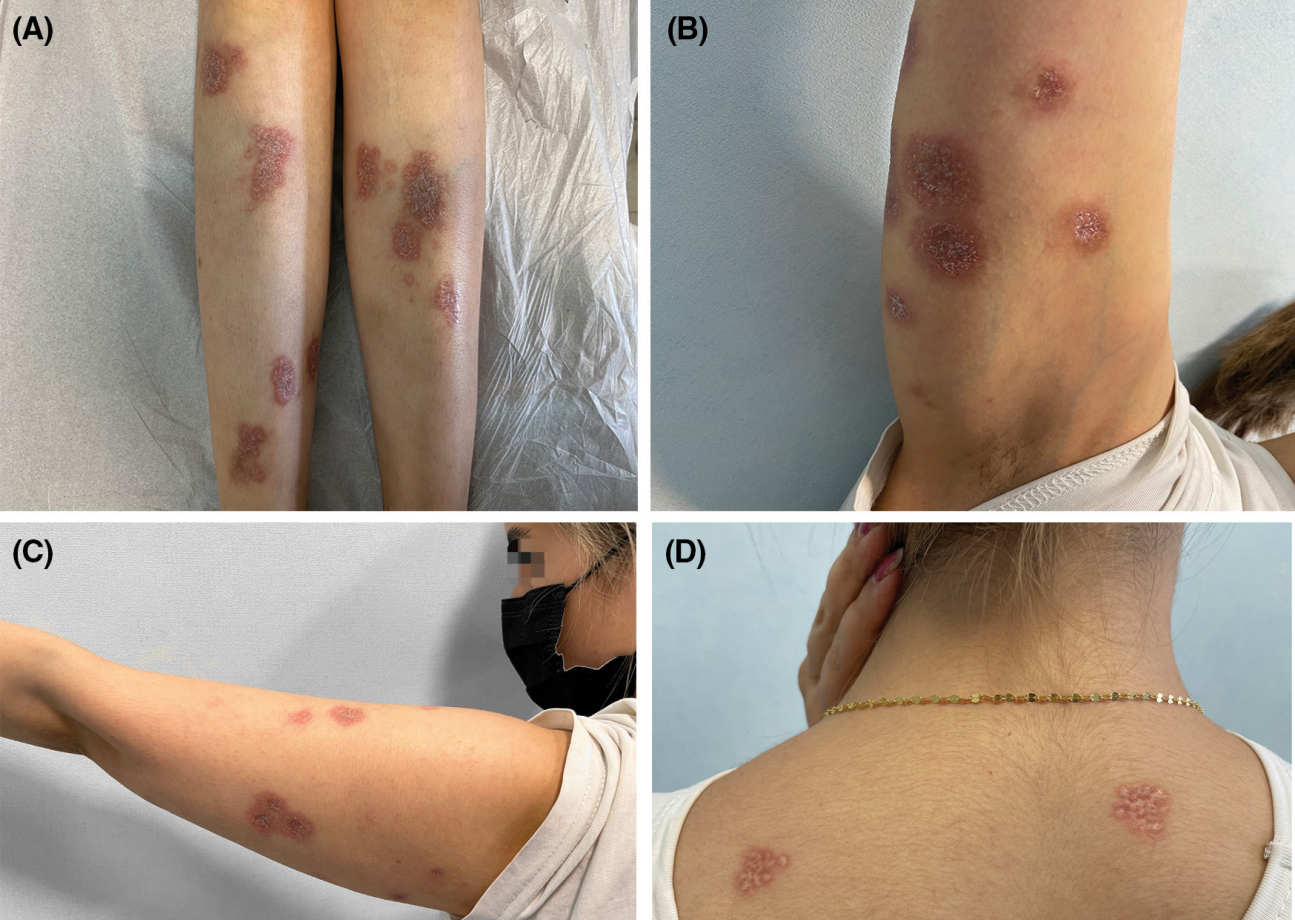
Clinical images. Multiple atrophic plaques involving patient's lower (A) and upper extremities (B,C). Similar lesions on the back (D).

A 25‐year‐old woman with type 1 diabetes mellitus was referred to a university‐affiliated dermatology clinic with a 2‐year history of disseminated cutaneous lesions. She was a known case of diabetes mellitus and hypothyroidism in the past 8 years and was taking insulin and thyroxine. During these years, she had uncontrolled diabetes. The skin lesions first appeared as erythematous patches on her legs (Figure 1A) and gradually spread to other parts of the body including upper extremities and back (Figure 1B–D). Clinically, the lesions consist of well‐demarcated yellow‐brown atrophic plaques and papules with comedo‐like openings and keratinous plugs.

**FIGURE 2:**
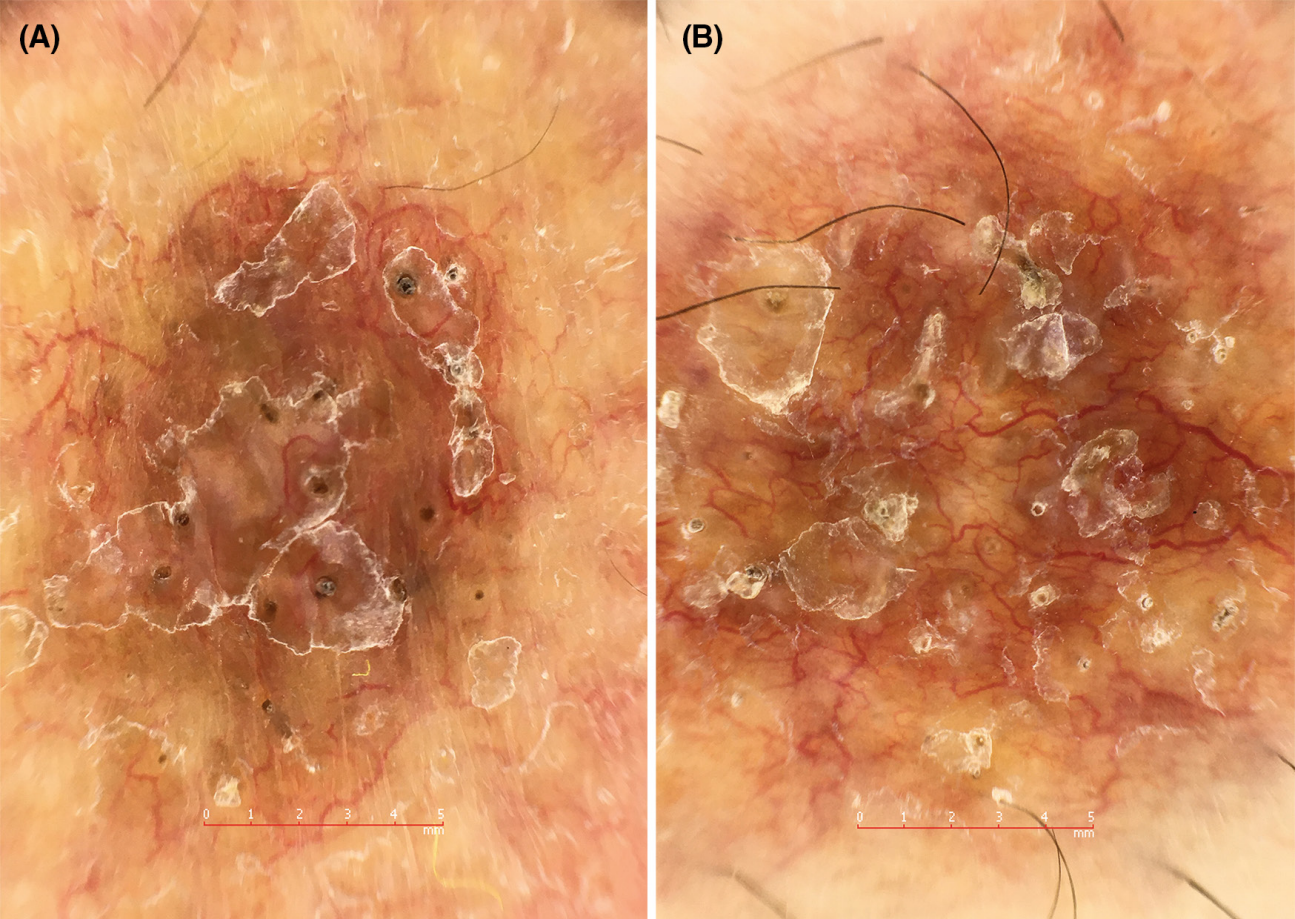
Dermoscopic images. Well‐focused linear vessels, comedo‐like openings, white structureless areas, follicular plugging, and silvery‐white lamellated scales on a pinkish‐yellowish background.

Dermoscopy revealed diffuse structureless yellowish‐pinkish background and well‐focused linear vessels. Additionally, there were comedo‐like openings that correspond to perforating degenerated collagen on histopathology. Scattered white structureless areas, follicular plugging, and silvery‐white lamellated scales were additional dermoscopic findings (Figure 2A,B).

**FIGURE 3:**
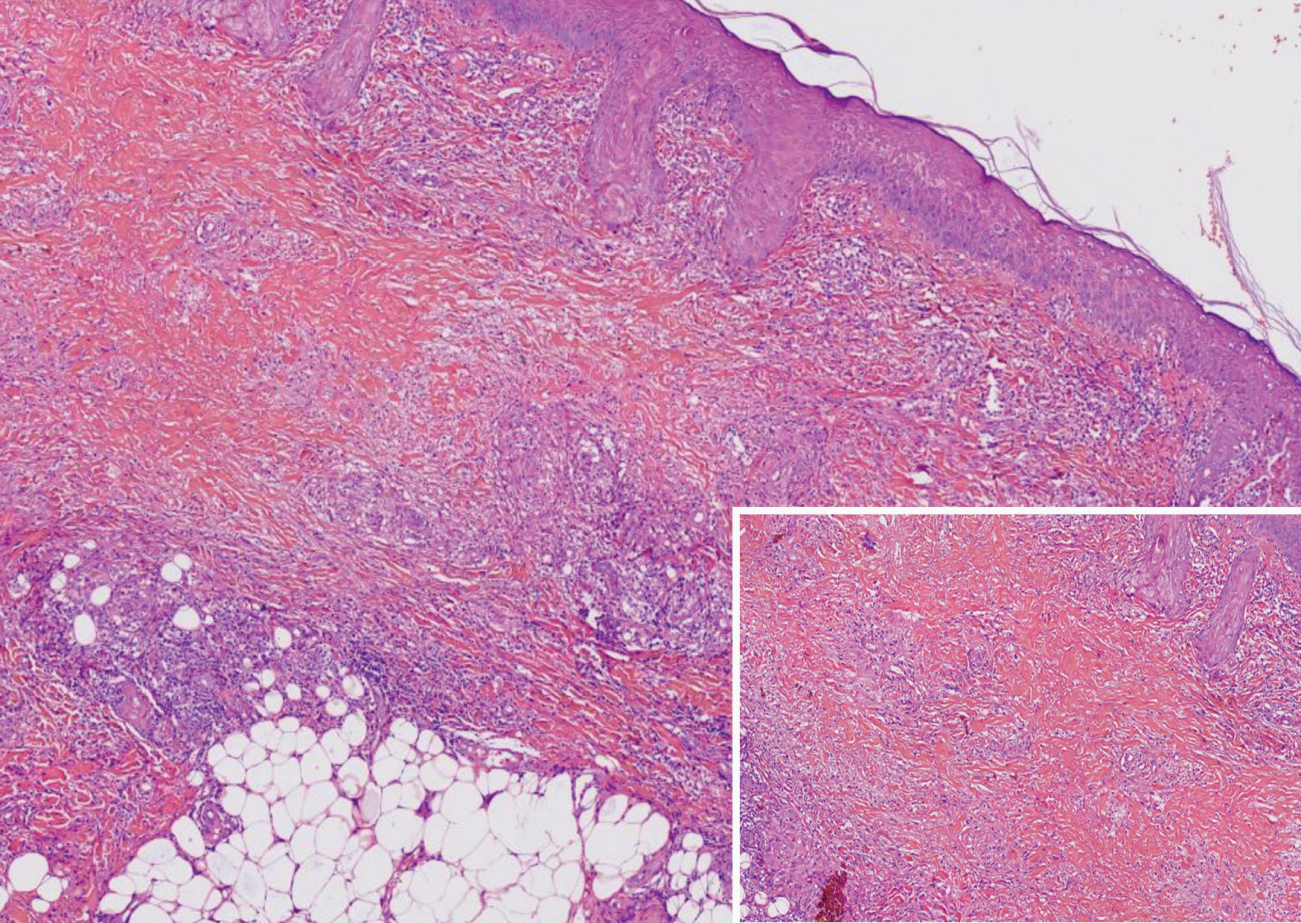
Histopathological images. Interstitial granulomatous dermatitis involving total dermis and subcutaneous tissue accompanied by areas of collagen degeneration with sclerosis. (H&E, ×40, and ×100 [inset]).

The biopsy specimen from the right arm showed interstitial granulomatous dermatitis involving total dermis and subcutaneous tissue accompanied by areas of collagen degeneration with sclerosis. These areas were surrounded by a variable lymphohistiocytic infiltrate (Figure 3).

The patient received topical potent corticosteroid for a duration of 1 year along with five separate injections of triamcinolone acetonide administered at monthly intervals. Unfortunately, these treatments did not yield any significant results. As the next step, we commenced a therapeutic trial by prescribing hydroxychloroquine at 400 mg daily for a period of 6 months. However, she did not exhibit any response to this medication and ultimately opted against pursuing further treatment.

## 3 DISCUSSION

Necrobiosis lipoidica is a rare granulomatous and inflammatory cutaneous disorder characterized by collagen degeneration associated with diabetes mellitus.^1^ Clinical manifestations of the perforating form include well‐demarcated yellow‐brown atrophic plaques with comedo‐like papules.^2^ Dermoscopy is a useful technique helping to identify perforating type of NL due to its ability to show transepidermal elimination of degenerated collagen as comedo‐like opening and keratotic plug.^8^ Most theories on pathogenesis revolve around microvascular damage. Microangiopathic vessel changes seen in diabetic patients could contribute to the development of collagen degeneration and subsequent dermal inflammation.^2^, ^5^ This altered material causes the expression of keratinocyte receptors for the specific types of collagen and elastin (e.g., collagen IV, V, VI, and 67‐kDa elastin receptor) resulting in the transepidermal and transfollicualr perforation of collagen, elastin, necrotic connective tissue, and calcium.^6^

Disseminated perforating NL represents an exceedingly very rare variant of NL. This form was defined as affecting at least the trunk and either the upper or lower, or both extremities.^9^ To the best of our knowledge, there have been only five previously reported cases of this variant. Our case is the sixth reported case of this variant; However, despite our patient, all previous cases were associated with type 2 DM.^4^, ^5^, ^6^, ^7^, ^8^ (Table 1).

**TABLE 1:** Reported cases of disseminated perforating necrobiosis lipoidica^a^.

Year of publication	Age/sex	Location of skin lesions	Histology	Dermoscopy	Treatment	Type of DM^b^	Ref. no.
1977	64 Male	Arms Chest Abdomen Left leg	Thin epidermis Dilated hair follicles with a plug of keratin and necrotic material Necrotic connective tissue intermingled with granulomatous infiltrate	–	–	2	4
2013	42 Female	Upper and lower extremities	Degenerated collagen and fibrosis Peripheral lymphohistiocytic infiltrate Transepidermal elimination of necrotic material	–	Topical steroid was ineffective	2	5
2015	69 Male	Upper and lower extremities	Multiple well‐defined collagen degeneration bordered by palisade of histiocytes Hyperkeratosis and elimination of necrotic collagen into dilated hair follicle infundibulum	–	Potent topical steroid ointment under occlusion with some improvement in cosmetic outcome	2	6
2018	49 Female	Chest Abdomen Extremities	Degenerated collagen being discharged outside through the hair follicle Palisaded granulomas with necrobiotic collagen in dermis	–	Tacrolimus ointment 0.1%, twice daily was ineffective	2	7
2020	60 Female	Buttock Upper and lower extremities	Granulomatous dermatitis involving the entire derma with areas of necrobiosis	Whitish/yellow background Irregular linear vessels Comedo‐like openings Hyperkeratosis	Methylprednisolone 30 mg/d for 3 months resulted in partial clinical improvement	2	8
Current case	24 Female	Back Upper and lower extremities	Granulomatous dermatitis and necrobiosis involving entire derma and areas of subcutaneous tissue	Yellowish/pinkish background White structureless area Sharp linear vessels Comedo‐like opening Follicular plugging Silvery‐white lamellated scale	Topical/intralesional steroids and hydroxychloroquine were ineffective	1	–

^a^ Defined as affecting at least the trunk and either upper or lower, or both extremities.

^b^ Diabetes mellitus.

As shown in Table 1, there were a total of six patients, including our case, with four being female and two being male. The mean age of patients excluding our case, was 56.8 years, which was significantly older than the current case. All patients had lesions on their extremities, and four patients (66.6%) had additional lesions on the chest, back, or abdomen. The histology in all cases showed collagen degeneration or granulomatous dermatitis with transepidermal and/or transfollicualr elimination.

Perforating NL is often resistant to conventional modalities such as topical or intralesional corticosteroids and immunosuppressive agents for stubborn lesions, and its management is a subject of debate.^2^, ^5^ It is evident that no treatment for NL has been proven effective in large randomized controlled trials (RCTs). However, the first‐line treatment is topical or intralesional steroid therapy. In cases where the disease is refractory to treatment, other options include topical photochemotherapy or systemic treatment with TNFα inhibitors, systemic steroids (preferably in nondiabetic patients), pentoxifylline, or hydroxychloroquine.^10^ Based on our literature review, unlike some localized forms, all cases of disseminated perforating NL including the current case were refractory to treatment. In light of this, dermoscopic examination helps not only to detect the perforating form of the disease but also becomes important from a prognostic point of view.

In conclusion, we present a case of disseminated perforating NL in a 24‐year‐old woman with type 1 diabetes mellitus. Diagnosis of perforating NL can be achieved through a clinical‐pathological correlation. However, dermoscopy provides additional clues for identifying the perforating type by examining the entire lesions and determining the best site for biopsy to observe the transepidermal elimination.

## AUTHOR CONTRIBUTIONS

**Mina Saber:** Conceptualization; data curation; resources; software; supervision; writing – original draft; writing – review and editing. **Fatemeh Mohaghegh:** Data curation; investigation; methodology; supervision. **Parisa Mohammadian:** Formal analysis; methodology; resources; visualization; writing – original draft.

## FUNDING INFORMATION

None.

## ETHICS STATEMENT

This study was performed according to the principles outlined by the World Medical Association's Declaration of Helsinki on experimentation involving human subjects and has been approved by the ethics committee of the Isfahan University of Medical Sciences (IR.ARI.MUI.REC.1400.062).

## CONSENT

Written informed consent was obtained from the patient to publish this report in accordance with the journal's patient consent policy.

## Data Availability

The data that support the findings of this study are available on request from the corresponding author. The data are not publicly available due to privacy or ethical restrictions.
